# Clinical study, network pharmacology, and molecular docking of Kunxian capsule in treating idiopathic membranous nephropathy

**DOI:** 10.3389/fmed.2025.1506972

**Published:** 2025-02-06

**Authors:** Jia Lv, Xinyu Gao, Lihua Liu, Libing He, Geng Tian, Xuehong Lu

**Affiliations:** ^1^Department of Nephrology, The Second Hospital of Jilin University, Changchun, China; ^2^Department of Gynecology and Obstetrics, The Second Hospital of Jilin University, Changchun, China

**Keywords:** Kunxian capsule, idiopathic membranous nephropathy, clinical study, network pharmacology, molecular docking

## Abstract

**Objective:**

A new *Tripterygium wilfordii* preparation called Kunxian capsule (KX) has been approved in China. However, it is still unknown whether KX is safe and effective for idiopathic membranous nephropathy (IMN) and its therapeutic mechanism of action is unclear.

**Methods:**

We conducted a retrospective study of 39 patients with IMN who received KX to investigate its efficacy and side effects of KX in treating IMN. We also used network pharmacology and molecular docking methods to explore the potential mechanism of action of KX in IMN.

**Results:**

In patients with IMN receiving KX treatment, 24 h urine protein was markedly decreased, whereas serum albumin levels increased. The overall clinical response rate was 79.49% after 6 months of treatment, and there were no significant adverse events. Quercetin, luteolin and kaempferol were the main bioactive ingredients of KX in treating IMN. AKT1, IL6, and TNF were core targets. The main potential mechanism of KX in treating IMN were pathways involved in cancer, the AGE-RAGE signaling pathway in diabetic complications, lipid and atherosclerosis. Molecular docking results showed that the binding force between the active ingredient and core target was relatively stable.

**Conclusion:**

KX is a safe and effective treatment option for IMN and can effectively improve serum albumin and 24 h urine protein levels in patients with IMN. This study preliminarily reveals the possible mechanism of KX in the treatment of IMN and provides a theoretical basis for future clinical research.

## 1 Introduction

Membranous nephropathy (MN) is an immune-mediated glomerular disease characterized by the deposition of immune complexes in the glomerular basement membrane and diffuse thickening. Approximately 20% of MN is secondary to autoimmune diseases, infectious diseases, tumors, drugs, or harmful substances and is therefore known as secondary MN. However, approximately 80% of MN still lacks a clear cause and is called idiopathic membranous nephropathy (IMN) (1). IMN is one of the common causes of adult nephrotic syndrome in China, accounting for approximately 20% of cases of primary nephrotic syndrome (2). Although spontaneous remission is a common feature of IMN, IMN has a one-third rule, that is, spontaneous remission in one-third of patients, persistent proteinuria in one-third, and progression to end-stage renal disease in one-third (3).

At present, IMN treatment is mainly based on immunosuppressive agents, such as calcineurin inhibitors, alkylating agents, or rituximab (RTX). However, serious adverse reactions often occur with these treatments, and the recurrence rate of IMN is high after discontinuation of calcineurin inhibitors. Therefore, the optimal management regimen for IMN remains controversial, and the current IMN treatment regimen should be optimized (4).

Traditional Chinese medicine has long been used to treat patients with kidney disease as an alternative therapeutic strategy (5). Preparations of *Tripterygium wilfordii* are well known for their immunosuppressive and anti-inflammatory properties and are frequently used to treat a variety of autoimmune diseases, including rheumatoid arthritis and Sjogren’s syndrome. Recently, they have also been used to treat glomerular diseases (6, 7). A new *Tripterygium wilfordii* preparation called Kunxian capsule (KX) has been approved in China. The traditional preparation of *Tripterygium wilfordii* has some limitations in clinical application, such as reproductive system damage, but KX can significantly lower reproductive toxicity through compatibility with traditional Chinese medicine (8). Studies showed that KX can inhibit kidney damage and T-cell infiltration, and had the potential to protect podocytes (9, 10). However, it is still unknown whether KX is safe and effective for IMN and its therapeutic mechanism of action is unclear. Therefore, we retrospectively described the response rate and side effects that occurred during treatment in 39 patients with IMN who received KX therapy, and explored the molecular targets and possible mechanisms of action of KX during IMN treatment based on network pharmacology.

## 2 Materials and methods

### 2.1 Clinical research

#### 2.1.1 Patients

Patients with IMN who visited the Second Hospital of Jilin University between March 2017 and March 2022 were enrolled in this study. The inclusion criteria were as follows: (1) age between 18 and 75 years; (2) pathological diagnosis of IMN (stages I–IV) confirmed by biopsy and treated with KX; and (3) patients who had complete information and were followed up for more than 6 months. The exclusion criteria were as follows: (1) concomitant treatment with immunosuppressants during KX therapy; (2) secondary nephrotic syndrome; (3) fertility needs or pregnancy; (4) life-threatening illnesses, other organ malfunctions, severe infections, or other serious conditions; and (5) incomplete follow-up.

#### 2.1.2 Treatment regimen

KX (Guangzhou Baiyunshan Chen Liji Pharmaceutical Factory Co., Ltd., 0.3 g/capsule) was administered orally three times daily at an initial dose of 0.6 g. After remission, the dose was gradually decreased to between 50% and 70% of the oral dose until it was stopped.

#### 2.1.3 Efficacy evaluation

Baseline and follow-up data were collected at 1, 3, and 6 months. The primary outcome was the disease response rate, including complete and partial responses. Complete response (CR) was defined as 24 h urinary protein quantification < 0.3 g; partial response (PR) was defined as 24 h urinary protein quantification > 50% decrease from baseline; all other cases were classified as non- response (NR). Any patient who achieved a CR or PR was considered successful.

#### 2.1.4 Statistical analysis

Statistical analyses were performed using IBM SPSS Statistics version 25.0. Continuous variables with a normal distribution are shown by mean and standard deviation as, $\bar{X}\pm S$ whereas continuous variables with a non-normal distribution are shown by median and interquartile ranges as *M*(*P*25, *P*75). Non-normally distributed data was log-transformed to normally distributed data before the statistical analysis. Indicators before and after treatment were compared using the paired sample *t*-test. To decrease the risk of Type I errors caused by multiple comparisons, Bonferroni correction was applied to adjust level of test. The figures were obtained using GraphPad Prism 9. The metrics that followed a normal distribution were represented using error bar, while the metrics that did not follow a normal distribution were represented using box plot. Statistical significance was defined as a *P* < 0.05.

### 2.2 Network pharmacology

#### 2.2.1 Acquisition of potential therapeutic targets of KX for IMN

KX is mainly composed of traditional Chinese medicines including *Lycii Frucyus*, *Cuscutae Semen*, *Epimordii herba*, and *Tripterygium hypoglaucum*. The bioactive ingredients of *Lycii Frucyus*, *Cuscutae Semen*, and *Epimridii Herba* were identified using TCMSP,^1^ and those of *Tripterygium hypoglaucum* were identified using HERB.^2^ The bioactive ingredients were screened by setting oral bioavailability (OB) ≥ 30% and drug-like properties (DL) ≥ 0.18 (11, 12). According to these bioactive ingredients, the corresponding targets were obtained from Uniprot^3^ (13). With “idiopathic membranous neopathy” as the keywords, the pathogenic targets of IMN were searched in GeneCards^4^ (14) and Online Mendelian Inheritance in Man (OMIM)^5^ (15). Finally, the above KX targets were intersected with IMN targets to obtain potential therapeutic targets of KX for IMN using the online Venn diagram tool.^6^

#### 2.2.2 Construction of the ingredient-target-disease network

The potential therapeutic targets and the bioactive ingredients were input into Cytoscape 3.9.1^7^ and free nodes were removed to establish an ingredient-target-disease network and screen out the main bioactive ingredients of KX for IMN.

#### 2.2.3 Construction of protein–protein interaction network

Potential therapeutic targets were submitted to the STRING platform^8^ with a confidence score of > 0.40. The PPI network was visualized using Cytoscape 3.9.1, and the core targets were identified.

#### 2.2.4 Enrichment analysis

Common targets of KX and IMN were uploaded to the DAVID database,^9^ and gene ontology (GO) functional enrichment and Kyoto Encyclopedia of Genes Genomics (KEGG) pathway analyses were performed. The results were visualized using an online bioinformatics tool.^10^

#### 2.2.5 Molecular docking verification

The structures of the ingredients with the top three degree values in the ingredient-target-disease network were downloaded from the TCMSP. The protein structures of the top three core targets in the PPI network were downloaded from the PDB database.^11^ AutoDockTools1.5.6 and AutoDock4.2.6 were used to perform molecular docking to verify the binding affinity between the targets and the bioactive component of KX. Binding conformations were visualized using PyMOL 2.4.0.

## 3 Results

### 3.1 Baseline characteristics of patients

A total of 39 patients were included in the study. Of these, 13 patients had hypertension (33.33%) and 6 had diabetes (15.38%) (Table 1).

**TABLE 1:** Baseline characteristics of patients.

Characteristic	Value (*n* = 39)
Age	52.56 ± 11.40
Gender	
Male (n,%)	26(66.67)
Female (n,%)	13(33.33)
BMI	24.11 ± 3.10
Smoking (n,%)	15(38.46)
Drinking (n,%)	12(30.77)
Diabetes mellitus (n,%)	6(15.38)
High blood pressure (n,%)	13(33.33)

### 3.2 Changes in plasma albumin and 24 h urine total protein

**FIGURE 1:**
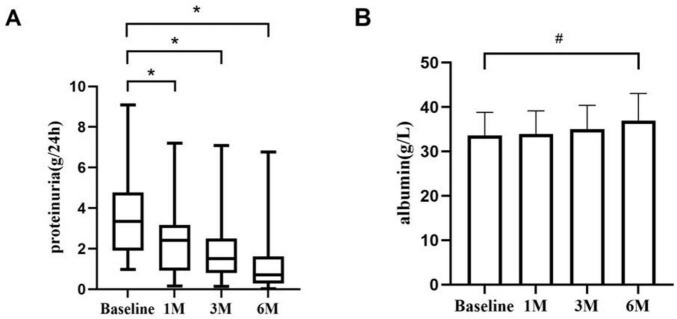
Proteinuria and serum albumin during KX therapy. **(A)** The proteinuria changes from baseline to 1, 3, and 6 months. There were significant differences in proteinuria at each time point. **(B)** The changes in serum albumin from baseline to 1, 3, and 6 months.*P < 0.01. #*P* < 0.05.

Baseline 24 h urine protein quantification was 3.34 (1.90, 4.77) g/d; at 1, 3, and 6 months of treatment, it fell to 2.40 (0.91, 3.17) g/d (*P* < 0.01); 1.50 (0.80, 2.50) g/d (*P* < 0.01); and 0.70 (0.28, 1.61) g/d (*P* < 0.01). After 6 months of treatment, the serum albumin increased from (33.63 ± 5.17) g/L at baseline to (36.99 ± 6.07) g/L (*P* < 0.05) (Figure 1).

### 3.3 Changes in other biochemical parameters

**FIGURE 2:**
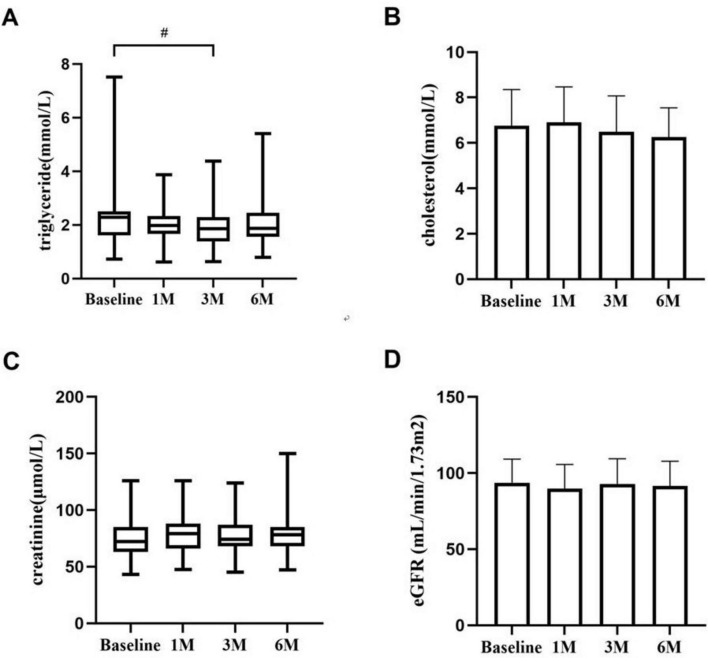
The changes in triglycerides **(A)**, blood cholesterol **(B)**, creatinine **(C)**, and eGFR **(D)** during KX therapy. #*P* < 0.05.

Changes in triglycerides and blood cholesterol levels during KX therapy are shown in Figure 2. Triglyceride levels in patients demonstrated a statistically significant difference at 3 months compared to baseline (*P* < 0.05). The serum creatinine level and eGFR of patients with IMN remained stable, and there was no significant difference at 1, 3, and 6 months (*P* > 0.05).

### 3.4 Response after KX therapy

**FIGURE 3:**
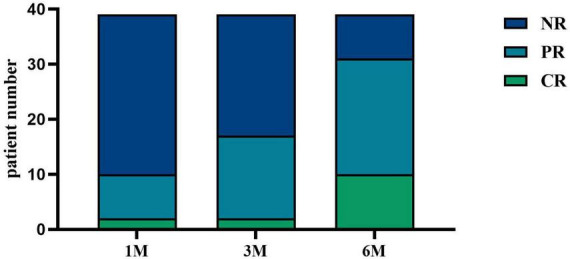
IMN response after KX therapy. CR was defined as 24-h urinary protein quantification < 0.3 g; PR was defined as 24-h urinary protein quantification > 50% decrease from baseline; all other cases were classified as NR. At 1 month, 5.13% (*n* = 2) of patients achieved CR, and 20.51% (*n* = 8) achieved PR. At 3 months, 5.13% (*n* = 2) of patients achieved CR, and 38.46% (*n* = 15) achieved PR. At 6 months, 25.64% (*n* = 10) of patients achieved CR, and 53.85% (*n* = 21) achieved PR.

After receiving KX therapy, 10 patients (25.64%) experienced remission at 1 month, and 17 patients (43.59%) experienced remission at 3 months. At 6 months, 31 patients responded, with an overall clinical response rate of 79.49%, including 10 patients with CR (25.64%) and 21 patients with PR (53.85%) (Figure 3).

### 3.5 Adverse events during KX therapy

Common side effects of KX included liver damage (17.95%), infections (including lung and urinary tract infections) (20.51%), increased creatinine levels (7.69%), and irregular menstruation (2.56%). Most adverse responses improved after symptomatic therapy. Although liver and renal function in some patients did not entirely return to normal at the end of the observation period, they did so during the follow-up period, and no significant fatal adverse reactions occurred.

### 3.6 Potential therapeutic targets of KX for IMN

**FIGURE 4:**
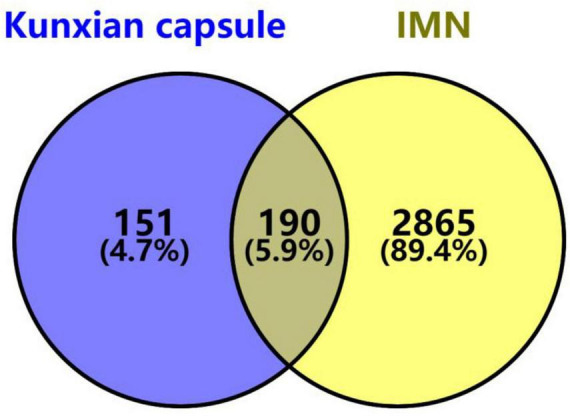
Venn diagram of KX targets and IMN targets. The purple section stands for KX targets, the yellow section stands for IMN targets, and 190 targets in the middle overlapping section are common targets of KX and IMN.

A total of 86 bioactive ingredients of KX were identified, including 45 from *Lycii Frucyus*, 11 from *Cuscutae Semen*, 23 from *Epimridii herba*, and 7 from *Tripterygium hypoglaucum*. After eliminating the duplicates, 81 bioactive ingredients of KX and 341 corresponding targets were identified. A total of 5581 targets of IMN were obtained from GeneCards, and 503 IMN targets were obtained from OMIN. A total of 3055 targets of IMN targets were obtained by integrating and removing the duplicates. A total of 341 targets of KX and 3055 targets of IMN were imported into the online Venn diagram tool to obtain 190 potential therapeutic targets (Figure 4).

### 3.7 Ingredients-targets-disease network

**FIGURE 5:**
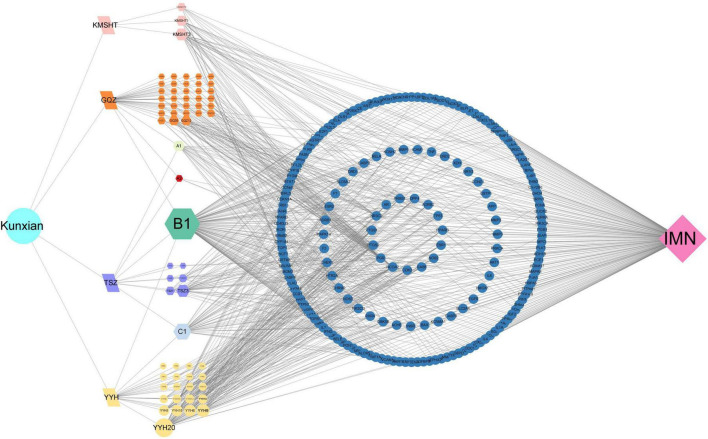
Ingredients of KX-targets-IMN network. The traditional Chinese medicine was represented by letters, “GQZ” for Lycii Frucyus, “TSZ” for Cuscutae Semen, “YYH” for Epimrdii Herba and “KMSHT” for Tripterygium hypoglaucum. The unique ingredients among them were represented by letters and numbers, such as “GQZ1,” and common ingredients were represented with A, B, C and numbers, such as “A1.”

The network comprised 67 bioactive ingredient nodes, 190 potential therapeutic targets, and 848 edges (Figure 5). According to the topological properties of the network, the larger the degree value of the node, the more targets it acts on and the more important the ingredient. The three main bioactive ingredients of KX were quercetin, luteolin, and kaempferol.

### 3.8 PPI network construction

**FIGURE 6:**
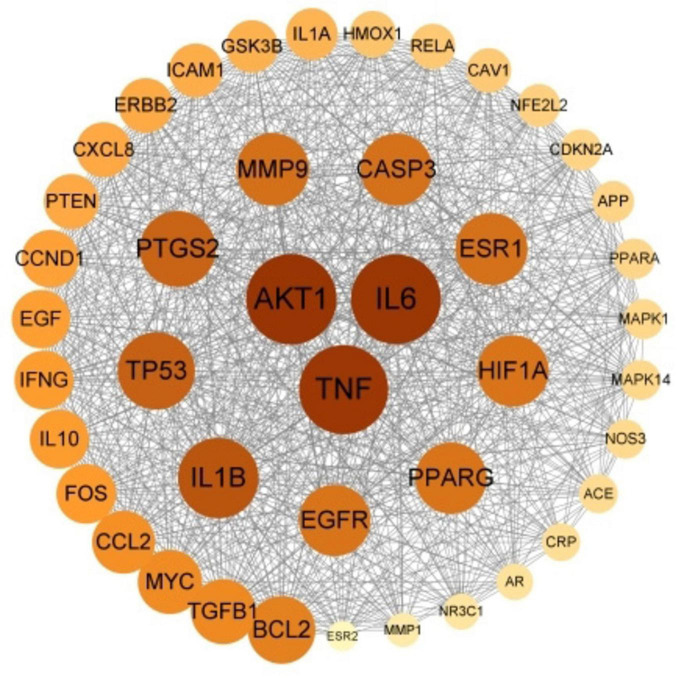
PPI network of KX targets against IMN. The color and the size of the nodes reflects the degree of connectivity (darker color and bigger circle indicates a higher degree).

The 190 potential therapeutic targets were uploaded to the STRING platform, and the results were inputted into Cytoscape 3.9.1. A total of 48 core therapeutic targets were screened using a betweenness centrality > 150, closeness centrality > 0.003, and degree centrality > 48. The top three core targets were AKT1, IL6, and TNF (Figure 6).

### 3.9 Possible regulatory signaling pathways of KX in treating IMN

**FIGURE 7:**
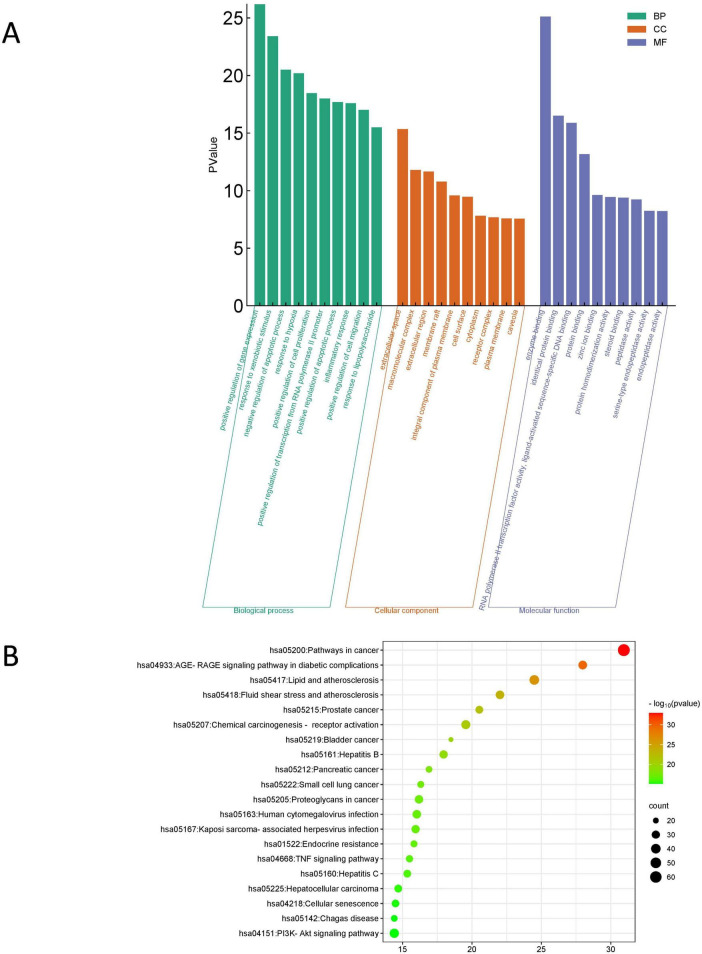
Enrichment analysis of the targets of KX in treating IMN. **(A)** GO functional analysis. The top 10 items of each part are shown. **(B)** KEGG pathway enrichment analysis. The sizes of the bubbles are illustrated from big to small in descending order of the number of potential targets involved in the pathways.

We further conducted GO functional analysis and KEGG pathway enrichment analysis to elucidate the biological effects on gene functions and signaling pathways of the related targets of KX in the treatment of IMN (Figure 7). In terms of biological processes, it was mainly related to positive regulation of gene expression and response to xenobiotic stimulus. The cellular components were mainly concentrated in extracellular space. For molecular functions, it can be seen that the targets were mainly enriched in enzyme binding. According to the results of the KEGG pathway enrichment analysis, it was most involved in cancer pathways, the AGE-RAGE signaling pathway in diabetic complications, lipid and atherosclerosis.

### 3.10 Molecular docking results

**FIGURE 8:**
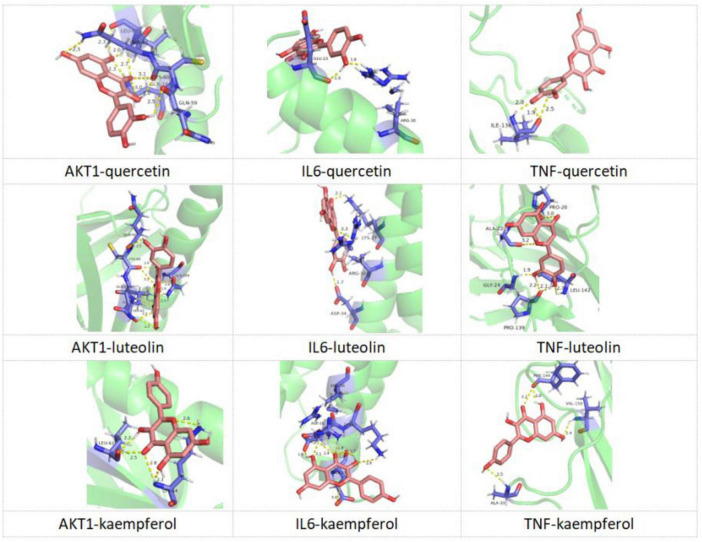
Molecular docking diagram of KX docking with core targets.

In the present study, the interactions between the core targets and KX were verified by molecular docking (Figure 8). It is generally believed that the lower the energy when the conformation of the ligand binding to the receptor is stable, the greater the possibility of interaction. Nine pairs were delivered to the docking simulation, and all binding complexes showed good binding affinity (Table 2).

**TABLE 2:** Molecular docking results of KX docking with core targets.

Ingredients	Targets	Energy (kcal/mol)
Quercetin	AKT1	−4.02
Quercetin	IL6	−2.72
Quercetin	TNF	−4.25
Luteolin	AKT1	−3.86
Luteolin	IL6	−3.04
Luteolin	TNF	−4.33
Kaempferol	AKT1	−3.42
Kaempferol	IL6	−3.56
Kaempferol	TNF	−5.12

The energy is an important parameter for evaluating the strength of the interaction between a receptor and a ligand. A negative value indicates that the binding is spontaneous, and the larger the absolute value, the more stable the binding.

## 4 Discussion

The treatment of IMN has long been a challenge. Although the Kidney Disease: Improving KDIGO guidelines recommend the use of glucocorticoids, cyclophosphamide (CTX), cyclosporin A (CsA), tacrolimus (TAC), and RTX as standard treatments for IMN, some studies have shown that the efficacy and safety of these drugs are unsatisfactory. For instance, glucocorticoids and CTX together have good clinical efficacy, but the frequency of non-fatal side effects is significant (16). Although calcineurin inhibitors can quickly produce remission, they have a considerable risk of relapse and nephrotoxicity (17, 18). As a new biologic drug, RTX has the potential to address disease etiology, cause illness remission, and stabilize and enhance renal function (19). However, RTX therapy is expensive, and approximately 40% of patients do not respond to it (20). Treatment has become much more challenging for some individuals who do not respond to these drugs and eventually develop refractory IMN. Therefore, further research is required to investigate IMN treatment alternatives.

Traditional Chinese medicine has been used in China to treat kidney diseases clinically, and it has demonstrated promising application possibilities with good efficacy and few side effects (21). *Tripterygium wilfordii* preparations are currently well-studied traditional Chinese medicine immunosuppressive agents and are widely used for the treatment of various autoimmune diseases because of their prominent anti-inflammatory and immunosuppressive effects (22, 23). KX is a new type of *Tripterygium wilfordii* preparation and has a good effect in the treatment of IgA nephropathy, rheumatoid arthritis, and ankylosing spondylitis (24, 25). According to our network pharmacology-based research, quercetin, luteolin, and kaempferol were the main bioactive ingredients of KX in treating IMN. AKT1, IL6, and TNF were core targets. The main potential mechanism of KX in treating IMN were pathways involved in cancer, the AGE-RAGE signaling pathway in diabetic complications, lipid and atherosclerosis. Quercetin, a flavonoid widely found in plants, exhibits anti-inflammatory and anti-injury effects (26). It can reduce the expression and activity of matrix metalloproteinase-2, thereby inhibiting the proliferation and migration of vascular endothelial cells (27). Luteolin is a plant-derived liver protective immunomodulator with diverse antioxidant, free radical scavenging, and anti-inflammatory activities (28, 29). Kaempferol is one of the main bioactive compounds in Kaempferia rhizome, with antioxidant/anti-inflammatory effects demonstrated in various disease models (30).

We discovered that KX therapy was helpful for IMN clinical remission in our clinical study. Therefore, we conducted a retrospective analysis of patients with IMN treated with KX at our center, evaluating the efficacy and safety of KX in 39 patients with IMN over 6 months. The results demonstrated substantial clinical benefits, with significant improvements in clinical indicators and a considerable proportion of patients achieving CR or PR, highlighting the potential utility of KX as a treatment option for IMN. One of the most striking outcomes was the progressive reduction in 24 h urinary protein levels over the 6-month treatment period. Baseline urinary protein levels, which averaged 3.34 g/d, decreased significantly to 0.70 g/d at 6 months (*P* < 0.01). Proteinuria is a major risk factor for disease progression and long-term renal damage, and this reduction reflects a remarkable improvement in glomerular barrier function. Clinically, this indicates a reduced risk of progression to chronic kidney disease (CKD). Serum albumin increased from a baseline of (33.63 ± 5.17) g/L to (36.99 ± 6.07) g/L at six months (*P* < 0.05), indicating a reduction in protein loss through urine and the restoration of oncotic pressure. These changes suggest the potential of KX to improve systemic homeostasis. Furthermore, the clinical remission rates also underscore the efficacy of KX. The improvements in lipid profiles, as well as the stability of creatinine and eGFR levels following KX treatment, represent additional benefits, enhancing its clinical utility beyond managing proteinuria and serum albumin. Second, nearly half of the patients with IMN were relieved by KX after 3 months of treatment, indicating that KX has a rapid onset of action and good efficacy.

The included patients with IMN in our study consisted of those with refractory membranous nephropathy who did not achieve satisfactory outcomes with standard treatments, as well as patients with underlying conditions such as hypertension and diabetes who experienced increased proteinuria. None of these patients used immunosuppressants concurrently during KX therapy.

Based on the study results, KX effectively reduced proteinuria and is a viable therapeutic option for patients with refractory membranous nephropathy who failed to respond effectively to standard treatments and could not continue their original regimen due to drug side effects or cumulative dosage limits. Additionally, for patients with long-term hypertension and diabetes, high blood pressure and hyperglycemia can damage the kidneys, increasing the risk of disease progression. According to some studies, hypertension and diabetes are factors contributing to disease progression in patients with IMN (31, 32). When these patients show significantly increased proteinuria, renal biopsy is necessary to clarify the cause of kidney function changes. In such cases, timely treatment of elevated proteinuria is essential. The Kidney Disease: Improving KDIGO guidelines currently lack standard treatment protocols for such patients, and who also do not meet the criteria for immunosuppressant use. KX has shown the ability to reduce proteinuria and slow the progression of kidney dysfunction in these patients.

Common adverse reactions of *Tripterygium wilfordii* preparations are hepatorenal toxicity, gastrointestinal reactions, reproductive system damage, and hematologic toxicity (7). KX is compatible according to the traditional principle of monarchy in Chinese medicine (33). It uses *Tripterygium hypoglaucum* as the monarch drug, and its active ingredient has anti-inflammatory and immunosuppressive effects. Epimrdii Herba is the minister drug, and its active ingredient can regulate immunity and gonadotropic function; combined with Lycii Frucyus and Cuscutae Semen, products of tonifying kidney and benefiting essence, it antagonizes the toxic effects of *Tripterygium hypoglaucum* on the reproductive system, but does not affect its original anti-inflammatory and immunosuppressive effects (25, 34).

In our study, some patients developed the above adverse reactions; however, they improved after active symptomatic treatment, and no fatal adverse reactions occurred. We observed no significant differences in albumin levels at 1 and 3 months of treatment compared to those before treatment. Since hypoalbuminemia is a common clinical manifestation in patients with IMN, it can lead to a hypercoagulable state, increasing the risk of thrombosis. Therefore, in clinical practice, for patients with IMN and low serum albumin levels, we recommend oral anticoagulant therapy to prevent thrombosis, when necessary, based on their venous thromboembolism risk assessment results. Additionally, some patients exhibited elevated liver enzymes early after treatment, which was considered to be related to the hepatotoxicity of KX. For patients who developed elevated liver enzymes during KX treatment, we administered oral hepatoprotective drugs, such as glutathione or silibinin, while regularly monitoring liver function. During the 6-month observation period, liver enzyme levels in the vast majority of patients returned to normal after receiving hepatoprotective treatment. It is important to note that although the common side effect of *Tripterygium wilfordii* preparations is reproductive toxicity, the incidence of gonadal suppression in this study was low and is considered to be related to the small number and older age of female patients. Although KX has reduced reproductive toxicity through drug compatibility, reproductive toxicity is still one of the main factors limiting its widespread application in specific populations. The current study on the reproductive toxicity of KX remains at a theoretical level, and more *in vitro* tests and in-depth studies are required.

One of the limitations of this study is its single-center retrospective design. All treatment options were jointly decided based on the experience of clinicians combined with the patient’s choice, which may lead to selection deviation; secondly, the poor follow-up data of patients in this center led to a small sample size and short observation time, which may bias the evaluation of the efficacy and safety of KX; thirdly, this study was a single-center study of patients in Northeast China, and the results may not be universal for patients with IMN nationwide and even worldwide. Therefore, additional prospective RCT trials with larger sample sizes and longer follow-up times are required to demonstrate the effectiveness and safety of KX in IMN.

## 5 Conclusion

KX is a safe and effective treatment option for IMN and can effectively improve serum albumin and 24 h urine protein levels in patients with IMN. This study preliminarily reveals the possible mechanism of KX in the treatment of IMN and provides a theoretical basis for future clinical research.

## Data Availability

The original contributions presented in the study are included in the article/supplementary material, further inquiries can be directed to the corresponding authors.

## Footnotes

1
http://old.tcmsp-e.com/tcmsp.php

2
http://herb.ac.cn/

3
https://www.uniprot.org/

4
https://www.genecards.org/

5
https://omim.org/

6
https://bioinfogp.cnb.csic.es/tools/venny/

7
http://cytoscape.org/

8
https://cn.string-db.org/

9
http://david.ncifcrf.gov

10
https://www.bioinformatics.com.cn/login/

11
https://www.rcsb.org/
